# STING signaling in inflammaging: a new target against musculoskeletal diseases

**DOI:** 10.3389/fimmu.2023.1227364

**Published:** 2023-07-10

**Authors:** Chenyu Song, Zhuoyi Hu, Dingjun Xu, Huihui Bian, Juan Lv, Xuanxuan Zhu, Qiang Zhang, Li Su, Heng Yin, Tong Lu, Yinghua Li

**Affiliations:** ^1^ Jiangsu CM Clinical Innovation Center of Degenerative Bone & Joint Disease, Wuxi TCM Hospital Affiliated to Nanjing University of Chinese Medicine, Wuxi, China; ^2^ Institute of Translational Medicine, Shanghai University, Shanghai, China; ^3^ Department of Orthopaedics, Wenzhou Hospital of Integrated Traditional Chinese and Western Medicine, Zhejiang, China; ^4^ Department of Critical Care Medicine, Changshu Hospital Affiliated to Nanjing University of Chinese Medicine, Changshu, China

**Keywords:** musculoskeletal diseases, aging, inflammation, cytoplasmic DNA, STING modulators

## Abstract

Stimulator of Interferon Gene (STING) is a critical signaling linker protein that plays a crucial role in the intrinsic immune response, particularly in the cytoplasmic DNA-mediated immune response in both pathogens and hosts. It is also involved in various signaling processes *in vivo*. The musculoskeletal system provides humans with morphology, support, stability, and movement. However, its aging can result in various diseases and negatively impact people’s lives. While many studies have reported that cellular aging is a leading cause of musculoskeletal disorders, it also offers insight into potential treatments. Under pathological conditions, senescent osteoblasts, chondrocytes, myeloid cells, and muscle fibers exhibit persistent senescence-associated secretory phenotype (SASP), metabolic disturbances, and cell cycle arrest, which are closely linked to abnormal STING activation. The accumulation of cytoplasmic DNA due to chromatin escape from the nucleus following DNA damage or telomere shortening activates the cGAS-STING signaling pathway. Moreover, STING activation is also linked to mitochondrial dysfunction, epigenetic modifications, and impaired cytoplasmic DNA degradation. STING activation upregulates SASP and autophagy directly and indirectly promotes cell cycle arrest. Thus, STING may be involved in the onset and development of various age-related musculoskeletal disorders and represents a potential therapeutic target. In recent years, many STING modulators have been developed and used in the study of musculoskeletal disorders. Therefore, this paper summarizes the effects of STING signaling on the musculoskeletal system at the molecular level and current understanding of the mechanisms of endogenous active ligand production and accumulation. We also discuss the relationship between some age-related musculoskeletal disorders and STING, as well as the current status of STING modulator development.

## 1 Introduction

The musculoskeletal system serves as the physical foundation for human morphology, stability, movement, and organ protection. With aging, the musculoskeletal system experiences a decline in muscle strength and size, decreased bone volume and quality, reduced cartilage thickness, and impaired intervertebral disc structural integrity. These alterations can lead to numerous physiological changes and disorders, including osteoporosis, muscle loss, increased fracture risk, osteoarthritis (OA), and intervertebral disc degeneration.

At the tissue level, aging of the musculoskeletal system is characterized by the development of chronic inflammation, known as inflammaging, which is linked to numerous age-related diseases. At the cellular level, the aging process involves the aging of related cells, the accumulation of senescent cells, the buildup of inflammatory factors, and metabolic imbalances. Cellular aging, represented by irreversible cell cycle arrest, can be classified into replicative senescence triggered by telomere shortening after a certain number of cell divisions (Hayflick limit) and premature senescence caused by various stressors (e.g., radiation, oxidative stress, activation of oncogenes) before reaching the Hayflick limit, also known as accelerated aging (1, 2). Telomere shortening and stress-related DNA damage not only trigger cellular aging but also lead to cytoplasmic DNA accumulation and activation of cGAS-STING signaling. The two direct functions of STING signaling, namely interferon-inducing and autophagy activities, are related to the inflammatory response and metabolic stability of aging cells.

Activation of STING plays a crucial role in defending against pathogen invasion and promoting anti-tumor immunity. For instance, STING activation can induce cellular senescence, promote dendritic cell maturation, and other mechanisms to exhibit anti-tumor effects (3, 4). Studies using STING agonists alone or in combination with other drugs have shown promising results, such as the use of STING agonist DMXAA in combination with chemotherapy drug SN38, which demonstrated strong anti-tumor effects in melanoma and colon cancer (5–7). However, prolonged or chronic inflammatory signals are key factors in the development of autoimmune diseases. Improper activation of STING, whether directly or indirectly, may lead to excessive inflammation and autoimmune diseases such as systemic lupus erythematosus and Acardi-Goutières Syndrome (8–11). Research has also found that the cGAS-STING pathway in innate immunity contributes to Alzheimer’s disease (12, 13). In recent years, blocking the STING signal has emerged as a potential therapeutic target for various musculoskeletal diseases. Achieving this goal necessitates comprehending the sources and mechanisms of early signals, such as cytoplasmic DNA, and advancing the development of STING-targeting drugs. This article reviews the impact of cGAS-STING signaling on the musculoskeletal system, the generation and accumulation mechanisms of endogenous ligands, the relationship between certain age-related musculoskeletal diseases and STING, and the current status of STING-targeting drug development.

## 2 Molecular mechanisms of cGAS-STING-mediated inflammation, metabolic disorder, and aging in the musculoskeletal system

**Figure 1:**
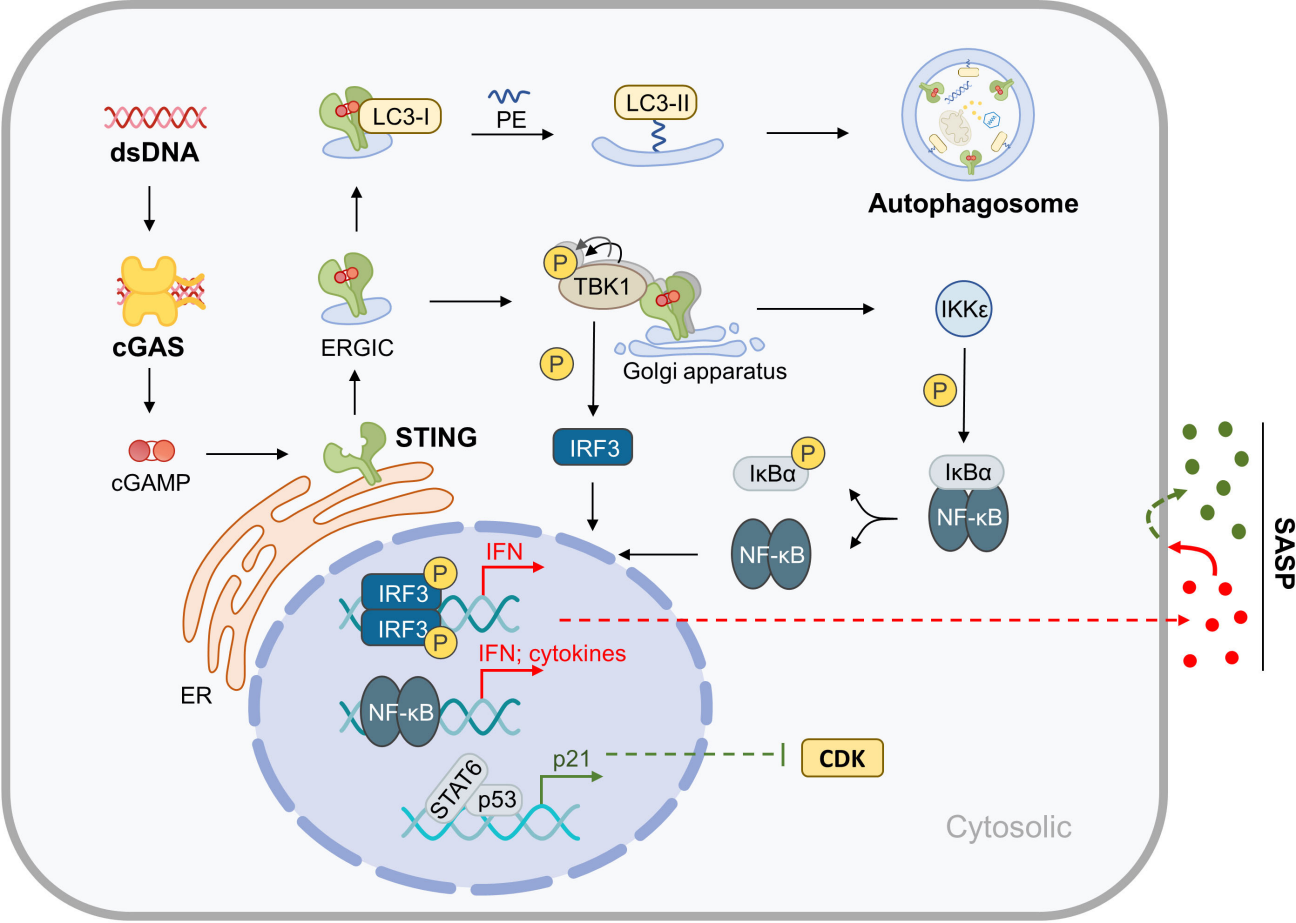
Molecular Mechanisms of cGAS-STING in Inflammaging. After binding with cytoplasmic dsDNA, cGAS catalyzes the synthesis of cGAMP, which is an active ligand of STING. Activation of STING results in the upregulation of autophagy and the expression of inflammatory factors, which in turn leads to SASP. Furthermore, the cGAS-STING pathway indirectly promotes cell cycle arrest.

STING is a transmembrane protein located in the endoplasmic reticulum (ER) that mediates the cytoplasmic DNA-induced cGAS-STING signaling pathway. This pathway involves the DNA sensor cGAS, the second messenger cGAMP, the adaptor protein STING, the kinases TANK Binding Kinase 1 (TBK-1) and IκB kinase (IKK), and downstream members. STING plays a central role in this pathway, and when it binds with cGAMP produced by cGAS, it drives two biological processes: Firstly, STING translocates to the Golgi apparatus for further processing and activation. During this process, the ER-Golgi intermediate compartment (ERGIC) carrying STING serves as the membrane source of autophagosomes, which may represent the more primitive function of STING in evolution (14). Secondly, activated STING binds and activates downstream kinases, initiating a cascade reaction that leads to the activation and nuclear translocation of transcription factors, such as NF-κB and IRF3. This upregulates the expression of genes such as type I interferons (IFNs) and inflammatory factors, which are directly upregulated by the cGAS-STING pathway. These factors upregulated by the cGAS-STING pathway can further cause multi-omics changes through autocrine and paracrine mechanisms, particularly the induction of the SASP related to aging. Furthermore, downstream members of STING can promote cyclin-dependent kinases (CDKs) inhibitor expression by regulating the expression or activity of certain transcription factors, thereby promoting cell cycle arrest (15–18). This section focuses on the molecular mechanisms of cGAS-STING-mediated inflammation, metabolic disorder, and aging in the musculoskeletal system (**Figure 1**).

### 2.1 STING and SASP in the musculoskeletal system

SASP has distinct effects depending on its duration. Under normal physiological conditions, short-term SASP recruits immune cells to clear senescent cells, leading to local immune responses that promote tissue repair and embryonic development (such as apical ectodermal ridge and neuronal development) (19, 20). However, during the aging process, a decline in immune function impairs the clearance of senescent cells that produce SASP, leading to the accumulation of senescent cells in tissues and sustained SASP factor production. This results in chronic inflammation and age-related diseases (21, 22), which are common in musculoskeletal aging and exist in almost all musculoskeletal diseases discussed in this article. Targeting the removal of senescent cells in the musculoskeletal system to block the production of SASP can alleviate the progression of these diseases.

The composition of SASP is complex and dynamic, including hundreds of protein and non-protein signaling molecules such as pro-inflammatory and immune-regulatory factors, chemokines, proteases, extracellular matrix (ECM), neurotrophins, DAMPs, etc. The specific composition varies spatiotemporally, depending on cell types and the triggering mode of aging (23), and also changes over time (24, 25). Some components are commonly present in different aging inducers and cell types (23), such as growth differentiation factor 15, stanniocalcin 1, and serine protease inhibitors, which are also aging biomarkers in human plasma and core SASP factors. There are also unique SASP factors in different tissues that play a key role in driving the progression of corresponding diseases, such as matrix metalloproteinases (MMPs) causing cartilage damage in OA.

Although the current understanding of SASP is incomplete, we know that SASP is associated with the DNA damage response (DDR) and involves various known or unknown biological processes. The cGAS-STING pathway is considered a key regulatory factor that induces SASP (26–28). For example, promoting the ubiquitination and degradation of STING can prevent the expression of SASP factors in aging joint cartilage (29). In the population, carriers of the STING R293Q allele may be less susceptible to aging-related diseases due to reduced inflammation (30).

The two main transcription factors activated through the STING pathway are IRF3 and NF-κB. As mentioned above, activation of STING leads to the activation of several kinases, such as TBK-1 and IKK. In the STING pathway that does not rely on cGAS-mediated activation of nuclear DNA damage, TRAF6 accumulates on STING, catalyzes the K63 polyubiquitination, and recruits TAB2/3 and TAK1, leading to the activation of the IKK complex (31, 32). Subsequently, the human NF-κB inhibitory protein α, phosphorylated by IKK, dissociates from the NF-κB dimer, allowing for NF-κB activation and translocation to the cell nucleus, which activates the transcription of pro-inflammatory cytokine genes, such as IL-6 and tumor necrosis factor (TNF).

Moreover, STING-related epigenetic regulation can act independently on the SASP, separate from its role in cell senescence. For example, in oncogene-induced senescence (OIS) cells, the H3K79 methyltransferase DOT1L is upregulated. DOT1L is downstream of STING and can cause H3K79me2/3 modification of the IL1A locus, which is an active histone mark that promotes IL1A expression. IL1A is a key upstream regulatory factor for other SASP-related genes (33).

This epigenetic regulation leads to the upregulation of SASP factors but does not induce cell senescence. Likewise, dual inhibition of H3K9me2 and H3K27me3 promotes tumor cell senescence. However, it is noteworthy that inhibiting these two methylations also reduces the production of cytoplasmic chromatin fragments (CCF), preventing the activation of cGAS-STING signaling and, therefore, avoiding SASP production (34).

### 2.2 STING and autophagy in the musculoskeletal system

In animals, autophagy is the process of delivering cellular material to lysosomes *via* autophagic pathways for further degradation. There are three main types: macroautophagy, microautophagy, and chaperone-mediated autophagy. In macroautophagy, a vesicle called an autophagosome first forms in the cytoplasm, sequestering a small portion of the cytoplasm containing soluble materials and organelles. The autophagosome then fuses with a lysosome to form an autolysosome, where the contents are degraded. In microautophagy, the lysosomal membrane directly invaginates to engulf small cytoplasmic components. In chaperone-mediated autophagy, substrate proteins are translocated across the lysosomal membrane with the help of chaperone proteins. Unless otherwise specified, in this article, autophagy refers to macroautophagy. Autophagy is a critical component of cellular metabolism, degrading foreign substances (xenophagy) and aged or damaged components into “building blocks” that can be reused in two ways: material turnover, such as protein synthesis, and stable energy production, such as providing metabolites for the TCA cycle and assisting in mitochondrial quality control (35, 36).

Basic-level autophagy is crucial for maintaining muscle and bone homeostasis, helping osteoblasts, osteoclasts, and chondrocytes respond to stress and nutrient deprivation (37). The intensity of autophagy needs to be within an appropriate range to maintain individual health, as both impaired and excessive autophagy can lead to diseases, aging, and shortened lifespan (38, 39). For example, impaired autophagy leads to typical aging phenotypes in muscles (40–42), while impaired or excessive autophagy both result in muscle atrophy and degeneration (43, 44).

The molecular coordination of the autophagy process involves five key stages (45) (1): the formation of a phagophore, also known as nucleation, is inhibited by mTOR. Factors such as hypoxia, low energy, and DNA damage can lead to a reduction in mTOR activity, inducing autophagy (2). Formation of the Atg5-Atg12-Atg16L complex, located on the outside of the phagophore membrane, recruits light chain 3 (LC3) for processing and maturation in the next stage (3). Microtubule-associated protein LC3 is processed into a lipidated form (LC3 lipidation) and inserted into the extending phagophore membrane, leading to the maturation of the autophagosome (4). The autophagosome captures the material to be degraded, which can be either random or selective (5). The autophagosome fuses with the lysosome membrane, forming an autolysosome, where a series of hydrolytic enzymes within the lumen degrade the contents.

STING plays a key role in regulating autophagy at various levels. Firstly, it facilitates autophagosome formation *via* non-canonical autophagy, whereby the LC3 protein is lipidated with phosphatidylethanolamine (PE) through a ubiquitin-like modification system involving Atg7 and Atg3. This results in the conversion of soluble LC3-I to the membrane-bound LC3-II form, which mediates phagophore elongation, autophagosome sealing, and selective uptake of substrates *via* p62 (46, 47). During this process, STING is activated and buds from the ER to the Golgi, where it serves as the membrane source for LC3 lipidation. The LIR motif of STING interacts directly with LC3 to mediate autophagy, bypassing upstream regulatory factors such as Atg9a and Beclin-1, but depending on downstream factors Atg5 and Atg16L (48, 49).

Secondly, STING induces autophagy through ER-Stress and the unfolded protein response (UPR) *via* its UPR motif. This triggers negative regulation of the AKT-mTOR pathway, leading to autophagosome formation. Additionally, STING interacts with the ER calcium sensor STIM1 to maintain calcium homeostasis (50, 51).

Finally, STING regulates autophagosome maturation *via* TBK1, which is responsible for autophagosome degradation. TBK-1 phosphorylates and modifies p62, increasing its affinity for ubiquitin chains (52). It also forms the NDP52-ULK-TBK1 complex, which drives xenophagy and is essential for autophagosome growth. Furthermore, TBK1 regulates microtubule dynamics and the cytoplasmic level of motor proteins, facilitating the transport of autophagosomes to the lysosomal-rich area (53). Autophagy also helps clear cytoplasmic DNA and mediates the signal attenuation of STING by degrading it. In the absence of TBK-1, autophagosome maturation is inhibited, and STING is not degraded by autophagy (52).

### 2.3 STING and cell cycle arrest in the musculoskeletal system

The progression of the cell cycle is dependent on Cyclins and Cyclin-dependent kinases (CDKs). Cyclins fluctuate periodically within the cell and combine with CDKs to form a complex. This complex binds to the promoter of the transcription factor SBF gene and promotes SBF transcription through RNA polymerase II phosphorylation. This, in turn, promotes the transcription of genes necessary for entering the next phase of the cell cycle (54).

Irreversible and permanent cell cycle arrest is a critical feature of cellular senescence in cells with proliferative potential. This phenomenon damages the stemness of stem cells, such as muscle stem cells (MuSCs) and mesenchymal stem cells, resulting in impaired regenerative capacity of tissues such as muscle and cartilage (55–58). DNA damage is the core factor responsible for cell cycle arrest, which, under the regulation of the genome quality control system in long-lived mammals, is effectively repaired and has lower levels of inflammation (59). Specifically, DNA damage or telomere shortening to a certain extent exposes telomere DNA, which triggers the DDR pathway (60). In the DDR pathway, the expression of the CDK inhibitors p16 and p21 is upregulated, leading to cell cycle arrest (61, 62).

This mechanism can prevent aging cells from passing on damaged genomes. However, unrestricted DNA homologous recombination can cause chromosomal rearrangements and chromosomal end-to-end fusion, allowing cells to bypass replicative senescence caused by telomere shortening and potentially develop into cancer cells. The cGAS-STING pathway functions more like a safeguard, promoting cells to follow the correct fate.

cGAS is distributed in both the cell nucleus and cytoplasm. The amount and activity of nuclear cGAS are restricted, and it does not function as a DNA sensor upstream of the STING pathway. Instead, it occupies unprotected telomeres and subtelomeres through protein-protein interactions and recruits CDK1 to DNA damage sites, inhibiting chromosomal end-to-end fusion by suppressing DDR signals in mitosis, thus promoting replicative senescence (63–65).

Once nuclear cGAS fails to prevent chromosomal end-to-end fusion, abnormal chromosomes will activate cytoplasmic cGAS-STING, promoting cellular senescence. DNA damage induces cell cycle arrest and also activates the cGAS-STING signaling pathway. STING can indirectly promote cell cycle arrest. STAT6 and p53 are transcription factors for the p21 gene (15). On one hand, STING-TBK1 induces STAT6 phosphorylation, leading to STAT6 nuclear translocation and activation of p21 transcription. On the other hand, IFN-α/β induces upregulation of p53 (16, 17), and the NF-κB subunit p65 also cooperates with p53 to mediate cell cycle arrest (18).

## 3 Production and accumulation of ligands that activate the cGAS-STING pathway

In the cGAS-STING pathway, the initial signal is received by cytoplasmic cGAS. It recognizes cytoplasmic double-stranded (ds) or single-stranded (ss) DNA with a double-stranded secondary structure and catalyzes the synthesis of the second messenger cGAMP, which triggers cGAS-STING pathway activation. Additionally, cGAS is also a potential RNA sensor that catalyzes cGAMP synthesis (66).

**Figure 2:**
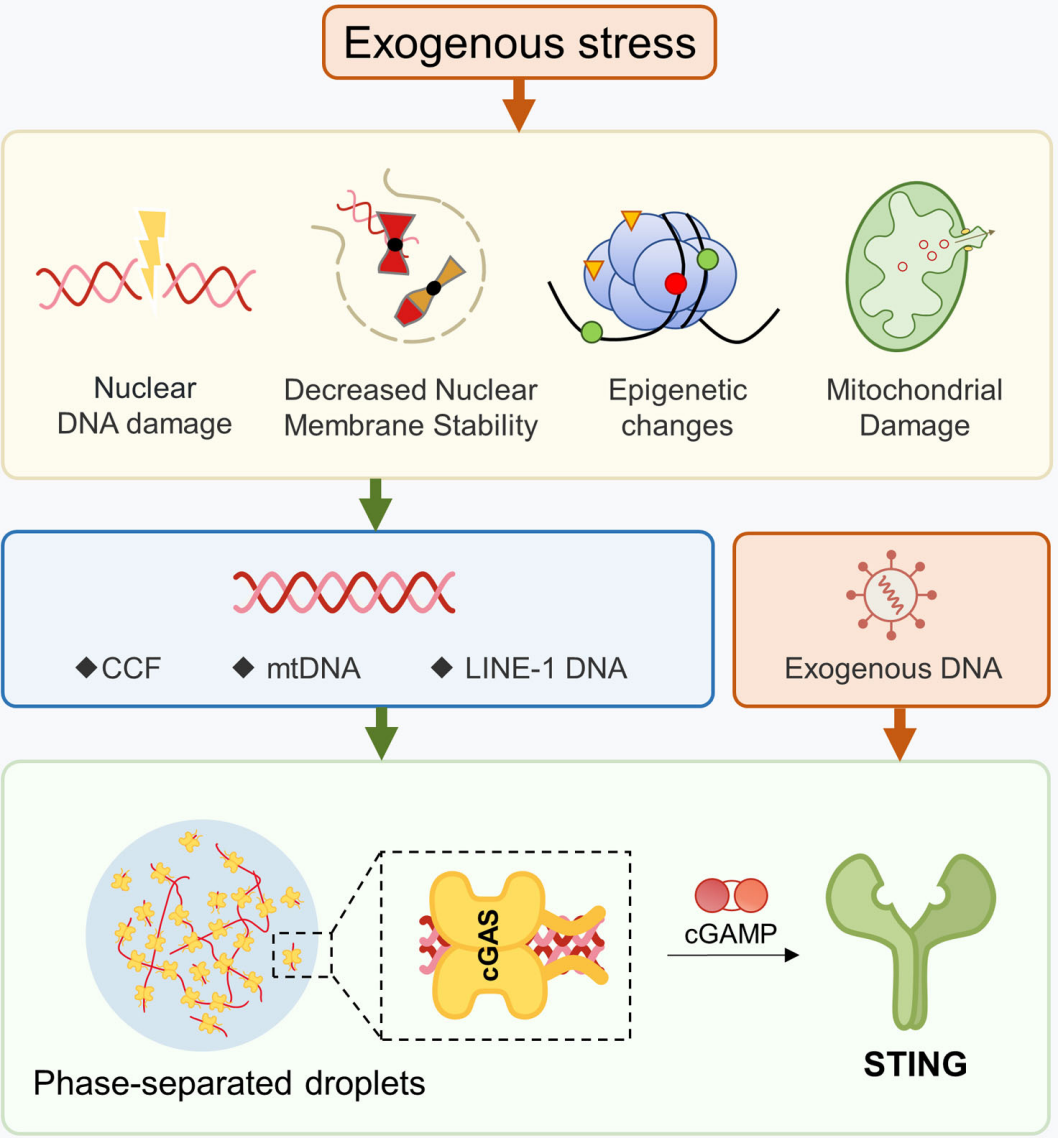
Production of Ligands for the cGAS-STING Pathway. Damage to DNA or mitochondria, as well as disruptions in nuclear membrane stability, can cause nuclear genes to escape into the cytoplasm. Changes in epigenetic modifications can un-suppress certain retrotransposons, leading to the presence of cytoplasmic dsDNA. After mitochondrial damage, mtDNA may enter into the cytoplasm. The formation of phase-separated droplets can hinder the ability of DNA enzymes to effectively hydrolyze dsDNA.

Ligand nucleic acids can be either exogenous or endogenous (67). In terms of viral infections, DNA viruses can directly activate cGAS, whereas RNA viruses can indirectly activate cGAS through mitochondria (68), which is beyond the scope of this article. Endogenous dsDNA can be further categorized into three types (1): nuclear DNA (2), mitochondrial DNA (mtDNA), and (3) retrotransposons. There are various mechanisms that lead to the accumulation of cytoplasmic DNA, such as telomere shortening, chromosomal damage, decreased nuclear membrane stability, mitochondrial dysfunction, and cytoplasmic DNA degradation obstacles. Although this article discusses them separately, it should be noted that these mechanisms are not single biological events during the aging process. They usually coexist and reinforce each other. For instance, in a cellular aging model with telomere shortening, the accumulation of dysfunctional mitochondria can cause mtDNA release and activate cGAS-STING (69). Aging cells with loss of nuclear membrane stability will undergo extensive DNA damage due to the nuclear entry of TREX1 (70). Oxidative stress can cause mitochondrial damage and nuclear DNA release, and oxidative modification of DNA makes it difficult to be effectively cleared by nucleases (71, 72). Furthermore, the STING and downstream signals can also be activated independently of cGAS (**Figure 2**).

### 3.1 Chromosomal DNA escaping from the nucleus

Genotoxic stress resulting from chromosomal damage can lead to the formation of micronuclei (MN). During the late stage of mitosis, damaged chromosomes may fail to properly attach to the spindle apparatus and separate correctly, resulting in their exclusion from the daughter nuclei during cell division. Instead, these chromosomes or chromosomal fragments exist in the cytoplasm within the nuclear envelope (NE) as micronuclei. The NE of micronuclei is very fragile, and after mitosis, more than half of the micronuclei rupture and release cytosolic chromatin fragments (CCF), which can activate the cytoplasmic cGAS.

This process is mainly driven by telomeric DNA damage. Shortened telomeres and telomeric double-strand breaks can cause end-to-end fusion of chromosomes, resulting in the inability of sister chromatids to separate during mitosis (39, 69, 73). This leads to fragmentation of nuclear DNA, which is released into the cytoplasm in the form of micronuclei that spontaneously rupture and further activate cGAS-STING (74). Repair of telomeric damage is unique compared to other parts of the genome since TRF2 and POT1 inhibit telomeric non-homologous end joining (NHEJ) to maintain genome stability and prevent end-to-end fusion of chromosomes (75).

DNA damage in other parts of the genome can also potentially induce micronuclei formation. Genotoxic stress can cause widespread DNA damage, which is generally repaired in vascular smooth muscle cells (VSMCs). However, telomeric DNA damage is prioritized in mediating the production of micronuclei and subsequent activation of cGAS-STING (75). This suggests that telomeric damage may be the primary driver of micronuclei formation in certain disease models where DNA repair capacity is normal. However, when DNA repair capacity is compromised, significant chromosomal DNA breaks and release into the cytoplasm can occur even without external stress. For example, in cells deficient in the androgen receptor (AR), DNA repair proteins Ku70 and Ku80 are not anchored to RNA Pol II, leading to dsDNA breaks, CCF leakage into the cytoplasm, and activation of STING (76). This study did not examine the contribution of telomeres and other parts of the genome to CCF release. Still, it is speculated that CCF here primarily arises from general genomic DNA breaks since RNA Pol II does not catalyze transcription of telomeres.

Besides micronuclei generated from chromosomal damage, CCF is also directly related to the reduced stability of the nuclear membrane in aging cells. Degradation of nuclear lamina protein B1 (Lamin B1) is recognized as a critical step in aging development. During aging, Lamin B1 is continuously degraded, causing uneven changes in the mechanical properties of the nuclear membrane, leading to the formation of nuclear blebs and even transient rupture under the influence of mechanical force and other factors (77). In aging cells processed by radiation and autophagy-lysosome pathways, CCF buds from the cell nucleus and is associated with the downregulation of Lamin B1 and loss of nuclear membrane integrity. Nuclear membrane damage can induce DNA damage and promote premature aging. Studies have found that nuclear membrane rupture can induce the relocation of the nucleotidyl transferase TREX1 to the cell nucleus, resulting in TREX1-dependent DNA damage (70, 78).

Both nuclear membrane stability and chromatin stability are essential for the proper functioning of a cell. For instance, in ionizing radiation-induced premature aging of human fibroblasts, not only a gradual loss of nuclear membrane stability was observed but also extensive recombination of chromatin occurred. Radiation stimulation affected various aging-related pathways, such as DNA damage or telomere stress-induced aging, chromatin organization, and senescence-associated heterochromatin foci (SAHF) aggregation (78). Conversely, mitigating DNA damage and preventing the degradation of nuclear lamina protein laminB1 downregulates cGAS-STING and downstream pathways activation (79). B1 downregulation is a common characteristic observed in aging, and it is considered one of the typical aging markers. Moreover, overexpression of B1 has been reported to induce cellular aging (80). In summary, the loss of nuclear membrane stability and the consequent damage to its integrity create a favorable environment for chromatin DNA release into the cytoplasm, which can lead to nuclear DNA damage.

The nuclear export pathway, which depends on the nuclear pore complex, may also contribute to the origin of cytoplasmic DNA. CRM1 is a critical protein that mediates nuclear export, and studies have reported that in aging cells treated with the CRM1 inhibitor leptomycin B (LMB), there is a significant reduction in CCF. Researchers suggest that the nuclear export pathway blocked by LMB partially overlaps with the expulsion of nuclear DNA (81).

### 3.2 Retrotransposons and epigenetics

Changes in epigenetic modifications of DNA and histones can activate the cGAS-STING pathway through retrotransposons. In keratinocytes with low DNA methylation, LTR retrotransposons, MLV retroviruses, and LV30 transposons are activated, leading to mitotic defects, micronuclei formation, cGAS-STING activation, and inflammation (82).

Proteins that maintain heterochromatin can also regulate cGAS-STING by controlling the activation of long interspersed nuclear element-1 (LINE-1), a retrotransposon that is often silenced in young cells. Once LINE-1 is derepressed, the resulting DNA can appear in the cytoplasm and activate cGAS-STING, thereby promoting cellular aging (83). For example, the transcription-translation feedback loop (TTFL) drives the oscillation of circadian genes, where BMAL1 acts as a transcription factor to initiate TTFL and interacts with heterochromatin-associated proteins and NE components. In the absence of BMAL1, the circadian rhythm is disrupted, and LINE-1 is no longer suppressed, leading to accelerated cellular aging through cGAS-STING activation (84).

Similarly, the histone deacetylase SIRT7 forms a complex with nuclear lamina proteins and components of heterochromatin to maintain heterochromatin structure. Its expression is downregulated during the aging process in various cell types, leading to transcription and retrotransposition of LINE-1. This mechanism has been observed to mediate cGAS-STING pathway activation and the aging phenotype in human mesenchymal stem cell (MSCs) (85, 86). SIRT6 also inhibits LINE-1, and its deficiency can lead to cGAS-STING activation, increased ROS, susceptibility to oxidative damage, accelerated cellular aging, and impaired function (87, 88).

Clearance of LINE-1 RNA is also critical. RNase H2 is necessary for effective LINE-1 retrotransposition, and its absence leads to upregulation of interferon-stimulated genes (ISGs) dependent on cGAS-STING and increased DNA damage. Researchers have proposed a model in which RNase H2 degrades LINE-1 RNA after retrotranscription to allow retrotransposition to complete (89). If RNase H2 is missing, and retrotransposition cannot be completed, the remaining DNA may activate cGAS-STING and trigger an inflammatory response.

### 3.3 Mitochondrial dysfunction

Mitochondrial dysfunction can lead to the direct or indirect activation of cGAS-STING. Damaged mitochondria release mtDNA into the cytoplasm, which acts as one of the cGAS ligands. The escape of mtDNA associated with cGAS-STING is influenced by various factors.

Viral infections can exacerbate mitochondrial damage, particularly in aged mice with shortened telomeres. These mice are more susceptible to respiratory viruses such as Influenza A virus (IAV) and SARS-CoV-2, which can cause excessive inflammation and higher mortality rates. This is mainly due to the leakage of mtDNA and the abnormal activation of cGAS-STING induced by viral infection (69). Similarly, infection with the vaccine strain PDK53 of dengue virus serotype 2 (DENV-2) drives cGAS synthesis of cGAMP by releasing mtDNA (90). Mechanistically, mtDNA is typically packaged into nucleoid structures, and the loss of the mtDNA binding protein TFAM, which regulates nucleoid structure, leads to moderate mitochondrial stress. Abnormal mtDNA packaging promotes mtDNA escape into the cytoplasm, thereby activating cGAS-STING.

Metabolic imbalances can also trigger mtDNA release and activation of cGAS-STING-dependent type I IFN signaling transduction. For instance, functional inhibition of FABP5, a member of the fatty acid binding protein (FABP) family, in regulatory T cells (Tregs) leads to impaired lipid metabolism, which can trigger mtDNA release and cGAS-STING activation (91). Furthermore, the loss of NAD^+^ in neurons with AMT (ataxia telangiectasia-mutated) gene defects induces mtDNA release into the cytoplasm, STING activation, inflammation, and aging. Conversely, increasing NAD^+^ levels can prevent aging and SASP (92).

Cytokine induction can also increase cytoplasmic mtDNA levels. For instance, TNF can induce mitochondrial damage in human myeloid THP-1 cells and increase cytoplasmic mtDNA levels. After TNF treatment, cytoplasmic mtDNA binds to cGAS, and the absence of cGAS reduces interferon response, inflammatory cell infiltration, and joint swelling in the absence of an arthritis model mouse (93).

Changes in mitochondrial membrane permeability may be a key factor in allowing mtDNA escape. For example, bacterial endotoxin lipopolysaccharide (LPS) can activate the pore-forming protein Gasdermin D in endothelial cells, leading to the formation of mitochondrial pores and mtDNA escape (94). Similarly, an increase in mitochondrial ROS can cause Gasdermin D to bind to the mitochondrial membrane and form mitochondrial pores, thereby releasing mtROS (95). In healthy U2OS cells, mtDNA is located in the mitochondrial matrix. During apoptosis, BAX/BAK-mediated mitochondrial outer membrane pores enlarge, allowing the inner membrane to be squeezed out through the outer membrane pore. Once the inner membrane enters the cytoplasm, it can penetrate and release mtDNA (96).

Mitochondrial damage can also indirectly activate cGAS-STING through CCF, an intermediate step in the collapse of cellular homeostasis during the response to exogenous stress. The pathway involves stress stimulation → nuclear gene imbalance → mitochondrial dysfunction → nuclear gene damage → CCF formation. Specifically, under ionizing radiation or oxidative stress, nuclear-encoded oxidative phosphorylation genes are down-regulated, leading to mitochondrial dysfunction. Dysfunctioning mitochondria induce the production of ROS and further trigger the ROS-JNK signaling pathway, which drives CCF formation, inflammation, and tissue damage. The specific mechanism by which JNK induces CCF formation is currently unclear, but researchers have suggested that it may be related to 53BP1, which is a regulator of DNA double-strand break repair and inhibits CCF formation while directly binding to JNK (71).

### 3.4 Impaired cytoplasmic DNA degradation

Impaired degradation of cytoplasmic DNA is also a contributing factor in cGAS-STING activation. As previously mentioned, the accumulation of cytoplasmic DNA can be observed in various models of inflammation, aging, or tumor cells activated by cGAS-STING. Cytoplasmic dsDNA is currently recognized as a signal that activates the cGAS-STING signaling pathway. However, in normal physiological states, two DNA enzymes, DNase2 and TREX1, can target and remove nuclear DNA in the cytoplasm to avoid abnormal activation of cGAS-STING (97, 98).

TREX1 is a nuclear-exonuclease anchored to the ER and distributed near the nuclear border. It is induced by genotoxic stress to ensure rapid degradation and release of nuclear DNA before cGAS activation, protecting cells from inappropriate autoimmune reactions (99, 100). Although TREX1 alone is not sufficient to clear all cytoplasmic DNA, it may preferentially bind and degrade nucleosome-free DNA that is most likely to activate cGAS (101, 102). Following micronuclei formation, the ER invades and ruptures the micronucleus, and TREX1 subsequently localizes to the micronucleus and degrades it (98). Some studies have shown that nucleosome-bound DNA is not a suitable substrate for TREX1, and other enzymes may be required for TREX1 to degrade micronucleus DNA (72, 100, 101, 103).

TREX1 can play a protective role in the genomic stress response by preventing the activation of cGAS. However, there is still controversy over whether it is a DNA repair gene or not (104, 105). As mentioned in section 2.1, DNA damage caused by nuclear membrane integrity damage even depends on the nuclear translocation of TREX1 (70). Under normal physiological conditions, a series of aging-related molecules, including TREX1, are intricately regulated and responsible for protecting against inflammatory damage. However, in a pathological state and in an unstable intracellular environment, these molecules may exacerbate the situation.

DNase2 is a nuclear endonuclease located in the lumen of the lysosome. MN DNA or nuclear export-mediated nuclear DNA escape will be engulfed and cleared by the lysosome, and then hydrolyzed by DNase2 (101, 106). In cell models of stress induction and replicative senescence, decreased activation levels and downregulation of DNase2 expression, weakened ability of autophagic lysosomes to engulf CCF, and cGAS-STING activation have been observed (81, 107).

Overall, the functions of these two DNA enzymes complement each other, and there may be other enzymes or protein factors that help them degrade cytoplasmic DNA. Once the imbalance of nucleases occurs, accumulation of cytoplasmic DNA and activation of cGAS-STING can be observed.

How do nucleases stop working and cause improper activation of cGAS-STING? There are a few ways this can happen. First, substrate insensitivity can occur; for example, DNA may be modified by oxidation to resist degradation by TREX1 (72). Second, decreased amount and/or activity of enzymes can occur due to mutations that impair the activity of DNase2 or TREX1 (108–111), downregulation of DNase2 and TREX1 expression in aging cells (97), downregulation of TREX1 expression mediated by microRNAs (112), or epigenetic silencing of TREX1 mediated by DNA methylation (113). Third, enzymes cannot reach their substrates: non-specific cGAS-DNA, cGAS-cGAS interaction that drives the formation of cGAS-DNA phase-separated droplets, which can prevent TREX1 from degrading DNA in these droplets (114, 115).

### 3.5 STING activation independent of cGAS

The cGAS-STING signaling pathway has been extensively studied, with STING acting as the adaptor protein activated by binding to cGAMP from cGAS, which is the most typical scenario. However, there is also evidence that in certain situations, STING can be activated independently of cGAS. This is mainly because STING can directly bind to exogenous ligands, and cGAS is not the only upstream factor for STING. Furthermore, many of these mechanisms are not yet fully understood.

Other cell-derived cGAMP and microbial-derived CDS can serve as direct ligands for STING, as observed in various studies (116–118). Enveloped RNA viruses can activate STING and downstream signaling during membrane fusion, independent of cGAS (119). In response to BV infection, the production of IFN-λ1 has been observed to be dependent on STING but independent of cGAS (120). Ku70, a DNA sensor, translocates from the nucleus to the cytoplasm and forms a complex with STING to stimulate the production of IFN-λ1 after DNA transfection or DNA virus infection (121, 122). While IFN-λ1 belongs to the type III IFN, and there is no report demonstrating that Ku70 can induce the production of type I IFN (123), the induction of IFN-αβ by DNA vaccines is also dependent on STING but independent of cGAS (124). This suggests that there may be other DNA sensors that can activate STING in the absence of cGAS in the STING-dependent type I IFN response. Furthermore, after genomic DNA damage, DNA binding protein IFI16, DDR factor ATM, and PARP-1 mediate STING activation independent of cGAS (125). Therefore, we can infer that STING acts as a hub in the signal network for DNA sensing.

## 4 STING and age-related diseases in the musculoskeletal system

**Figure 3:**
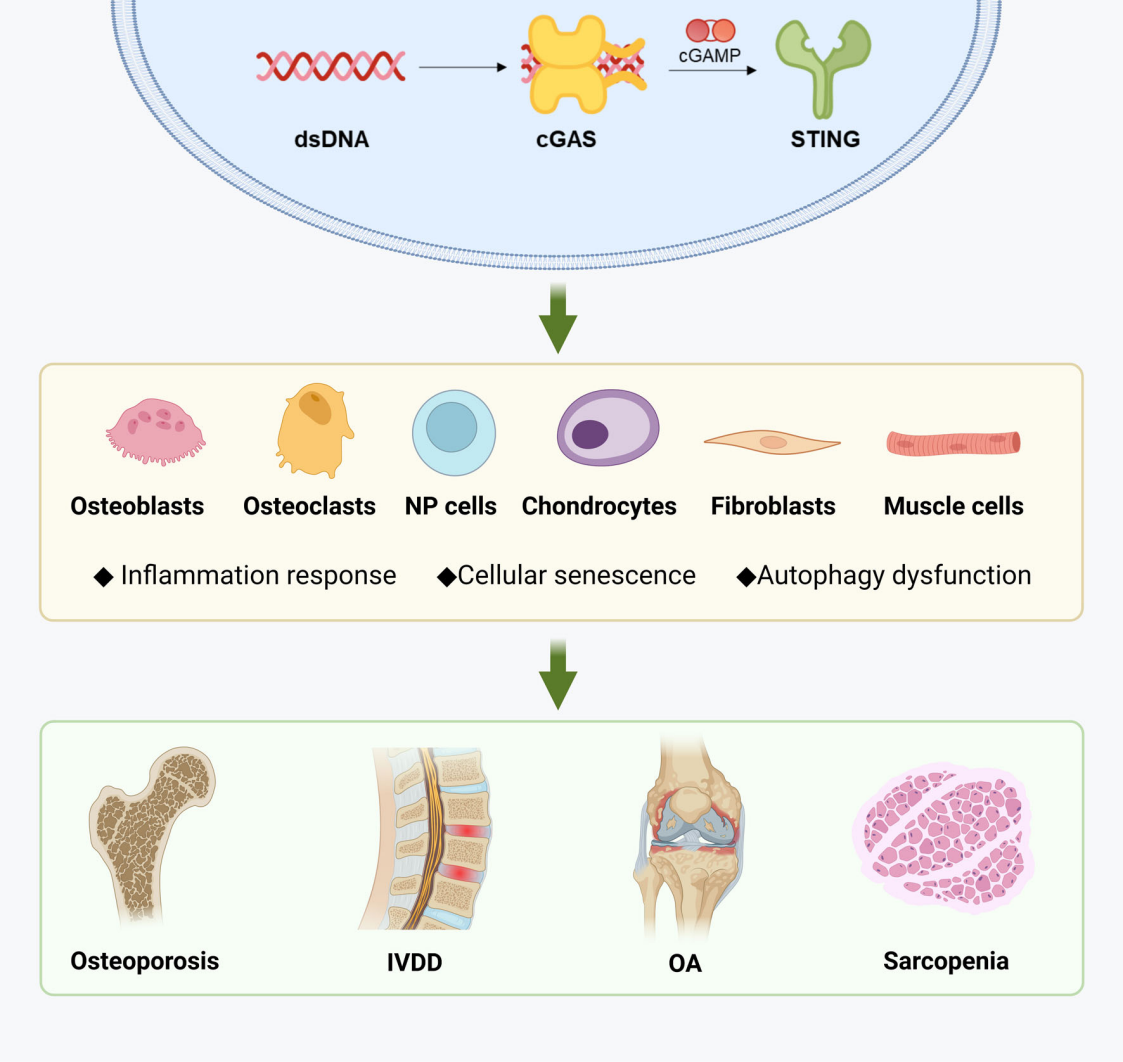
Diseases related to musculoskeletal aging and STING. Created with BioRender.com.

The STING pathway can be activated by cytoplasmic dsDNA resulting from DNA damage, leading to metabolic imbalance, inflammation, and ultimately promoting cell cycle arrest and inflammatory senescence. In the context of specific musculoskeletal diseases, STING is involved in maintaining the metabolic homeostasis of various cell types, with its activity exhibiting both commonalities and heterogeneities across different diseases at both the cellular and molecular levels. Briefly, the STING pathway has a complex impact on the generation and function of osteoclasts and osteoblasts, thus playing a role in the progression of osteoporosis and OA. STING activation also accelerates the degradation of ECM in OA and intervertebral disc degeneration by promoting the catabolism and senescence of chondrocytes and nucleus pulposus cells. While the mechanism of STING in muscle wasting syndrome is yet to be elucidated, it is closely related to the maintenance of muscle homeostasis. Overall, the precise role of STING in the musculoskeletal system warrants further investigation, and fine-tuning of regulatory pathways may represent a promising strategy for managing these diseases (**Figure 3**).

### 4.1 Osteoporosis

Osteoporosis is a condition characterized by a decrease in bone mass and an increase in bone fragility, which significantly increases the risk of fractures. Bones are composed of approximately 30% inorganic ions, 70% collagen and non-collagenous proteins, and minerals such as calcium, phosphorus, magnesium, sodium, and bicarbonate are maintained in balance by various cells. The formation of osteoporosis is related to osteoblasts (OBs), which form bone, and osteoclasts (OCs), which absorb bone. An imbalance between bone formation by OBs and bone resorption by OCs and changes in the quantity and function of bone marrow stromal cells (BMSCs), which are the precursor cells of OBs, can lead to osteoporosis.

The conditioned medium of senescent cells not only increases the formation of OCs but also impairs OB mineralization (126). BMSCs aging leads to a decrease in the number of OBs and reduces their potential for osteogenic differentiation relative to adipocytes, which results in a decrease in bone formation (127–131). Other cells in the bone environment also indirectly regulate bone mass through OBs and/or OCs activity. For example, megakaryocytes (MKs) can stimulate OBs proliferation, but their ability to regulate OBs function decreases with age, which may be one of the reasons for age-related bone loss (32).

STING regulates bone homeostasis in a bidirectional manner. Downstream factors of STING, such as IL1, IL6, and TNF-α (NF-κB pathway), induce the differentiation of OC progenitors, while other factors such as IFN-β (IRF3 pathway) downregulate the differentiation of OC progenitors (132). STING activation in humans and mice leads to significant IRF3/IFN-β signal transduction and relatively weak NF-κB activation (133, 134). At the same time, the activation of the NF-κB pathway, which is activated simultaneously, has a negative impact on the treatment of osteoporosis. The drug RTA-408 can inhibit STING-dependent NF-κB signaling and suppress mouse OC generation without affecting STING/IFN-β (135).

OBs are similar and regulated by cytokine networks such as IL-10 and IFN-γ, which promote osteoblastogenesis, while TNF-α, IFN-α, and IFN-β inhibit osteoblastogenesis (136). Although there is no research explaining the role of STING in OBs, it can be speculated that STING may be a potential target for regulating OBs differentiation. Downstream factors of STING, such as TNF-α, IFN-α, and IFN-β, are osteoinhibitory factors, but the relationship between STING and osteopromotive factors is much more ambiguous (137–139).

Autophagy plays a crucial role in regulating bone homeostasis. In OBs, autophagy suppresses oxidative stress-induced cell death and activates protective autophagy under low pH conditions (140, 141). Additionally, autophagy is involved in OBs mineralization and bone homeostasis regulation (142). In OCs, autophagy regulates differentiation and migration (143). However, the role of STING-mediated autophagy activity in these processes remains unclear.

In conclusion, the role of STING in osteoporosis is complex, as it is involved in maintaining a state of equilibrium among various factors. Precise regulation of signaling pathways may be key to treating such diseases.

### 4.2 Intervertebral disc degeneration

Intervertebral Disc Degeneration (IVDD) is a condition that affects the intervertebral discs (IVD) located between two adjacent vertebral bodies. These discs consist of nucleus pulposus (NP), annulus fibrosus (AF), and vertebral endplate (144). NP is a gel-like tissue that is highly hydrated and mainly consists of collagen, proteoglycans, and hyaluronic acid. The AF surrounds the NP and is connected to the upper and lower endplates to prevent NP overflow. When the spine moves, the nucleus pulposus deforms, which can absorb shock and buffer pressure.

IVDD can cause lower back pain, lumbar disease, and cervical disease. During the development of the disease, the ECM is degraded, the structure of the annulus fibrosus is damaged, and the cartilage endplate ossifies. Ultimately, IVDD can lead to protrusion of the intervertebral disc and a significant loss of height (145). The molecular mechanisms of IVDD are mainly related to NP cell aging, inflammation, and ECM degradation, which are induced by DNA damage (146–149).

STING may play an important role in IVDD and exhibits a dual role similar to regulating OCs. There is evidence supporting that STING activation promotes the progression of IVDD, while inhibiting STING can alleviate IVDD changes. For example, in degenerated NP tissue, ROS induces upregulation of STING expression in NP cells (144). Overexpression of TNF-α and STING both induce NP aging, apoptosis, and ECM degradation, while inhibiting NP cell aging and STING-TBK1/NF-κB and IRF3 signaling activation can suppress ECM degradation and improve the progression of IVDD (150, 151). There is crosstalk between the NF-κB pathway and autophagy, and inhibition of NF-κB not only has anti-inflammatory effects but also upregulates autophagy (152). Upregulation of autophagy also inhibits NF-κB, thereby suppressing IL-1β-induced NP cell apoptosis (153).

Of the two direct functions of STING, it is clear that the inflammatory activity of STING needs to be suppressed in the treatment of IVDD, while the upregulation of autophagy can improve IVDD. Exercise, certain endogenous hormones, or drugs can activate autophagy to protect NP cells from aging and apoptosis, and inhibit ECM degradation (154, 155). As described in the first section of this article, autophagy mediates STING signal attenuation. Studies have found that metformin can deactivate cGAS-STING signaling through autophagy and also inhibit NP cell aging (156). However, excessive autophagy is also a detrimental factor in IVDD and needs to be suppressed to provide protective effects (157, 158). Currently, research on IVDD rarely considers the effect of STING’s autophagy activity, but the two do not conflict in treatment because their interferon-inducing activity and autophagy activity can be partially uncoupled, and autophagosomes are not influenced by downstream factors. Therefore, in STING-targeted therapy, autophagy factors also need to be considered. This suggests that even for drugs and methods targeting the same disease and the same target, individual differences in patients need to be considered in precision medicine, otherwise it is highly likely that drugs and methods that have significant therapeutic effects in certain populations will worsen the condition in other similar populations.

It is important to note that the role of STING in IVDD is not well-established and continues to be a topic of debate. A study has suggested that the cGAS-STING signal does not contribute to the aging and degeneration of intervertebral disc cells during systemic inflammation, and no significant alterations were observed in IVD of mice with functional mutations or deletions of STING (159). This study implies that systemic inflammation may not be a critical factor in the development of IVDD.

### 4.3 Osteoarthritis

Articular cartilage comprises chondrocytes and ECM, which play a crucial role in reducing friction between bones, aided by synovial fluid. The collagen fiber network and proteoglycans can absorb pressure and vibration. OA is a degenerative joint disease characterized by joint pain and limited joint movement.

In a steady state, articular chondrocytes usually remain in a quiescent state (reversible cell cycle arrest) with low levels of proliferation (160). However, in the early stages of OA, chondrocytes proliferate in “clusters” to repair damaged cartilage matrix. Nevertheless, such chondrocytes are more prone to cellular senescence (161, 162). As the sole cells that generate and maintain the cartilage matrix, chondrocyte and MSC cell cycle arrest directly impede cartilage repair (55, 56). Moreover, the production of SASP leads to OA-related pathological changes (163), such as inflammation, cartilage matrix degradation, subchondral bone remodeling, synovitis, and OA pain (164, 165). The senescence of chondrocytes is the cause of OA, whether it is age-related OA or post-traumatic OA, hindering the repair of damaged cartilage and promoting various pathological changes (166–168).

There are multiple types of cells present in the synovium, including synovial fibroblasts that secrete synovial fluid to lubricate joints, while synovial macrophages secrete pro-inflammatory and anti-inflammatory factors to regulate the microenvironment stability. Aging synovial fibroblasts exhibit impaired autophagy and upregulated SASP, whereas enhancing their autophagic flux and inhibiting the generation of SASP factors can improve surgical-induced OA model in mice with damaged cartilage (169). It is currently unclear whether the SASP of the synovium comes from synovial fibroblasts or macrophages.

Subchondral bone is the bone layer beneath the cartilage, maintained by OBs and OCs to maintain bone remodeling homeostasis in response to mechanical load. Pathological changes in subchondral bone are one of the key factors in OA, even occurring earlier than cartilage damage and osteophyte formation (170). In this process, OCs generation increases, bone resorption is enhanced, causing chondrocyte hypertrophy, differentiation, and MMP synthesis, and bone and marrow-like cells undergo senescence and secrete SASP, which is associated with bone loss (171).

Similarly to osteoporosis, OCs and OBs contribute to the progression of OA by participating in the bone remodeling process and osteophyte formation beneath the cartilage. Currently, it is unclear how STING affects OC and OB in the joint microenvironment of OA patients and the resulting effects. However, the effects of STING on the metabolism and cell cycle of other joint cells are relatively clear.

The leading cause of OA is DNA damage, which triggers the overexpression and activation of STING in chondrocytes. This, in turn, stimulates the expression of enzymes that degrade cartilage matrix, such as MMP13 and ASAMTS4, through the NF-κB pathway. Additionally, STING activation inhibits the production of components that make up the cartilage matrix, including aggrecan and type II collagen, and induces cell aging and apoptosis (172). Conversely, blocking the STING-dependent NF-κB pathway can decrease inflammation, synovitis, cell aging, and cartilage degradation in chondrocytes (173). Our research also supports this finding, demonstrating that targeting the proteasome to promote STING ubiquitination and degradation can improve age-related and trauma-induced OA (29). A recent study demonstrated that STING not only influences the pathological changes of cartilage and subchondral bone in osteoarthritis (OA), but also functions as a critical regulator of OA mechanical sensitivity. Inhibition of STING can effectively alleviate OA-associated mechanical allodynia (174).

Despite ongoing efforts, identifying optimal strategies for reversing joint disease progression remains challenging. Nevertheless, inhibiting STING has demonstrated promise in reducing inflammatory factors and preserving and repairing the cartilage matrix in animal models of arthritis.

### 4.4 Sarcopenia

Skeletal muscle is composed of bundles of muscle fibers that contract and relax to facilitate bodily movements in humans and other vertebrates (175, 176). Sarcopenia, a common condition among older adults, is characterized by a decline in muscle mass and function, increasing the risk of falls, fractures, disability, and death (177, 178). While senescent cells have not been identified in adult skeletal muscle, tissue damage can activate MuSCs for regeneration and generate senescent muscle cells (178–181).

Although research on the relationship between STING and skeletal muscle is scarce, we hypothesize that STING may play a role in maintaining muscle homeostasis. Firstly, according to the Human Protein Atlas (www.proteinatlas.org), STING is present in various muscle tissues. Secondly, factors that activate STING, such as telomere shortening, mitochondrial damage, oxidative stress, and DNA damage, also cause muscle wasting (182–184). Thirdly, excessive autophagy or damage can reduce muscle mass and function, and the correct turnover of substances is necessary to maintain muscle homeostasis (179, 185). Additionally, muscle energy homeostasis is maintained through the TCA cycle and oxidative phosphorylation (186, 187). And the SASP, particularly pro-inflammatory and pro-fibrotic factors, appears to play a crucial role in muscle wasting (188, 189). Mechanistically, in aging muscle cells, the upregulation of NF-κB, IRF3, and interferon pathways creates an inflammatory microenvironment that induces senescent cell accumulation and impairs MuSC proliferation. The pro-inflammatory and pro-fibrotic SASP factors appear to have a direct causal relationship with NF-κB (180).

However, it is not clear whether STING has an impact on muscle strength and quality changes, and what kind of effects it has. Further research is needed to confirm these questions.

## 5 Agonists and Inhibitors of STING

STING plays a crucial role in the cGAS-STING immune signaling pathway, which mediates the signaling of multiple DNA receptors and is expressed on the surface of several cell types, including dendritic cells, macrophages, and fibroblasts. STING is widely expressed in multiple cell types of the tumor microenvironment, where it triggers the balanced secretion of type I interferon and pro-inflammatory cytokines. Its activation not only stimulates T cell proliferation to kill tumor cells, but also enhances the release of tumor-associated antigens (190). Additionally, STING-mediated signaling pathways are closely associated with autoimmune diseases (8, 191), inflammatory diseases (192), neurological diseases (193–195), and metabolic diseases (196–198).

Therefore, targeting STING has emerged as a promising approach for the development of novel therapies for various diseases, including cancer and inflammatory diseases. Biopharmaceutical companies such as Merck Sharp & Dohme (MSD), Bristol-Myers Squibb (BMS), GlaxoSmithKline (GSK), Bayer, Novartis, and many others are involved in drug development targeting STING.

### 5.1 Agonists of STING

Several human STING agonists are being developed, including cyclic nucleotides (CDN) and non-nucleotides. CDNs (199) are natural agonists of STING, but their large molecular mass and high polarity make it challenging for them to pass through cell membranes. Moreover, their phosphodiester bond is easily hydrolyzed by enzymes and is metabolically unstable, limiting their biological activity and medical applications. To overcome these limitations, chemical modification based on the natural structure is an effective means of improving the medicinal properties of CDNs, and CDN analogs are synthesized by modifying the phosphate, ribose, and base sites of CDN. Non-nucleotide agonists can avoid the deficiencies of CDNs and are suitable for industrial production with low preservation costs.

However, some agonists have failed clinical trials because they only bind and activate murine-derived STING (mouse STING, mSTING) and do not bind human STING (hSTING) (200–202). Additionally, over-activation of STING can lead to sustained cytokine production, causing uncontrollable inflammation, cytokine storm, tissue toxicity, autoimmunity, and an inflammatory tumor microenvironment that promotes tumor growth (203). Therefore, the dose of STING agonists needs to be accurately controlled to maximize efficacy while minimizing immunotoxicity. The current development of STING activator products in the clinical stage is focused on five directions: CDNs small molecules, non-cyclic dinucleotide small molecules, Antibody drug conjugate (ADC), small molecules + vectors, and engineered bacteria. **Table 1** displays the STING agonists currently in clinical development.

**Table 1:** STING agonists in the development process.

Drug Name	Category	Therapy Area	Status
ADU-S100	CDN	Cancer	Phase II (Termination)
MK-1454	CDN	Cancer	Phase II (Termination)
GB492	CDN	Cancer	Phase I/II
BMS-986301	CDN	Cancer	Phase I
E7766	CDN	Cancer	Phase I
SB11285	CDN	Cancer;Infection	Phase I
GSK3745417	Non-CDN	Cancer;Infection	Phase I
TAK-676	Non-CDN	Cancer	Phase I
SNX281	Non-CDN	Cancer	Phase I
HG381	Non-CDN	Cancer	IND
CRD-100	Non-CDN	Cancer	Pre-clinical
CRD5500	Non-CDN	Cancer	Pre-clinical
TTI-10001	Non-CDN	Cancer	Pre-clinical
AN3005	Non-CDN	Cancer	Pre-clinical
HH18202	Non-CDN	Cancer	Pre-clinical
XMT-2056	ADC	Cancer	Phase I
TAK-500	ADC	Cancer	Phase I
exoSTING	CDN+exosome	Cancer	Phase I/II
SYNB1891	Engineered Bacteria	Cancer	Phase I

### 5.2 Inhibitors of STING

While the STING signaling pathway is crucial for defending against DNA pathogens, excessive activation can result in an imbalance, leading to autoimmune and neurological diseases (191, 204–208). To address this, researchers have developed selective small molecule inhibitors that target STING, such as C-176, C-178, C-170, C-171, and H-151. These inhibitors covalently bind to Cys91 in STING proteins, preventing palmitoylation induced by STING activation, blocking its assembly into multimeric complexes in the Golgi apparatus, and inhibiting downstream signaling pathway transduction (209).

Additionally, the small molecule SN-011 and its derivatives have also been found to inhibit the STING signaling pathway by binding specifically to the pocket where STING proteins bind to their endogenous ligand molecule 2’3’-cGAMP (210). More recently, researchers have developed protein hydrolysis-targeted chimeras (PROTACs) as a novel drug discovery strategy. Chen et al. (211) reported the first STING-targeting PROTAC, which degraded STING activity and had a good anti-inflammatory effect in a cisplatin-induced acute kidney injury model in mice.

The development of STING inhibitors offers potential therapeutic options for autoimmune and neurological diseases. The commonly used STING inhibitors are listed in **Table 2**.

**Table 2:** STING inhibitors in the development process.

Drug Name	Category	Mechanism of action	REF
C-178 C-176 C170, C-171	Nitrofurans	Binds to Cys91 of STING and prevents palmitoylation	(184)
H-151	Indole derivatives	Binds to Cys91 of STING and prevents palmitoylation	(184)
SN-011	Multisubstituted benzamides	Specific binding within the 2'3'-cGAMP conjugation pocket of STING protein	(185)
Astin C	Cyclopeptides	Directly regulates STING signaling vesicles and binds to the C-terminal structural domain of STING	(186)
degrader 55	PROTACs	connecting the sting inhibitor C-170 to a ligand for E3 ligase CRBN through an alkyl linker	(187)

### 5.3 Inhibitors of STING in bone-related degenerative diseases

In addition to their role in inflammatory and autoimmune diseases, STING proteins have been implicated in the pathogenesis of senescence-associated degenerative diseases (194). Aging is a complex process characterized by chronic low-grade inflammation, which drives aging-associated diseases. Bone degenerative diseases often coexist with these aging-associated diseases. As aging progresses, there is an increase in senescent cells in the bone microenvironment, leading to increased production of senescence-associated pro-inflammatory secretory proteins, resulting in increased bone resorption and decreased bone formation. Inhibition of the STING signaling pathway has become a new target in the fight against musculoskeletal disorders, as elimination of senescent cells or inhibition of the production of pro-inflammatory secretory proteins delays the onset or inhibits the severity of many chronic diseases. Recent studies have found that a variety of inhibitors that directly or indirectly target STING show promising results in the treatment of bone-related degenerative diseases.

#### 5.3.1 STING inhibitors promote angiogenesis and accelerate bone healing

H-151 (209) is a highly selective small molecule inhibitor that covalently binds to Cys91 in STING proteins, blocking palmitoylation induced by STING activation, which in turn blocks its assembly into multimeric complexes in the Golgi apparatus and inhibits downstream signaling pathways. Chen et al. (212) demonstrated in a fracture or bone defect mouse model that H-151 promotes angiogenesis and osteogenesis at low doses. This suggests that inhibition of STING enhances H-type angiogenesis and accelerates the process of bone healing.

#### 5.3.2 STING inhibitors inhibit the production of OCs

RTA-408 is a synthetic triterpene compound currently under clinical investigation for various diseases. Sun et al. (213) used an ovariectomy (OVX)-induced bone loss model in C57BL/6 mice to show that RTA-408 inhibits osteoclastogenesis and attenuates OVX-induced bone loss by inhibiting the STING-dependent NF-κB signaling pathway, suggesting that it may be a promising candidate for the future treatment of osteoporosis. CDNs, such as cyclic diadenosine monophosphate and cyclic digluconate monophosphate, are symbiotic bacterial-derived second messengers in the intestine. In recent years, CDNs have been found to regulate immune activity in macrophages by inducing type I interferon expression through the STING signaling pathway. Kwon et al. (214) found that CDNs inhibited the differentiation of bone marrow-derived macrophages into OCs by inducing phosphorylation of TBK1 and IRF3 in a dose-dependent manner. Experiments in a mouse cranial implant model showed that CDNs inhibited Rankl-induced bone resorption. These results suggest that CDNs inhibit OCs differentiation and bone resorption by inducing IFN through the STING signaling pathway.

#### 5.3.3 Inhibitors of STING prevent the development of an inflammatory microenvironment in the nucleus pulposus tissue and slow disc degeneration

Intervertebral disc (IVD) degeneration is a prevalent musculoskeletal degenerative disease characterized by progressive nucleus pulposus (NP) cell death and the development of an inflammatory microenvironment in NP tissue. Excessive accumulation of cell membrane DNA, a damage-associated molecular pattern (DAMP), triggers immune responses in many degenerative diseases through the cGAS-STING axis (215). Zhang et al. (216) demonstrated that oxidative stress activates the cGAS-STING axis and NLRP3 inflammasome-mediated thermal degeneration in a STING-dependent manner. Using a rat disc pinning model, they found that the STING-specific inhibitor H-151 effectively reduces NLRP3 inflammasome-mediated NP cell death and microenvironmental inflammation *in vitro*, slows degenerative skeletal progression, and provides a promising therapeutic approach for disc-derived degenerative diseases.

#### 5.3.4 STING is implicated in articular cartilage degeneration

Cartilage is an important component of the joint and loss of articular cartilage remains a major factor in joint dysfunction. It has been shown that Gelsemium elegans-derived Gelvirine treatment inhibits IL1-β-induced chondrocyte expression of pro-inflammatory factors as well as STING and p-TBK1, and increases the expression of anti-inflammatory factors. In animal models, Gellelsevirine treatment attenuates age-related medial meniscus induced OA destruction. However, Gellelsevirine treatment did not have a protective effect on chondrocytes in a STING-deficient model. Further exploration of the mechanism suggests that gellelsevirine degrades STING *via* the K48 polyubiquitination (Lys48) pathway, thereby ameliorating age-related and surgery-induced OA in mice (29).

## 6 Conclusion

The STING pathway is an important mechanism regulating the immune response and has been implicated in the development and progression of a number of autoimmune diseases and viral infections. The cGAS-STING signaling pathway is activated by cytoplasmic dsDNA, which originates from various sources such as chromosomal DNA released from the nucleus, retrotransposons, and mtDNA. Genotoxic stress, retrotransposon activity, and mitochondrial dysfunction are some of the factors that contribute to cytoplasmic dsDNA accumulation. In skeletal muscle-related diseases, which include a variety of pathophysiological processes such as muscle atrophy, muscle weakness and fibrosis. Excessive activation of the STING pathway may lead to inflammatory responses and cell death, thereby affecting muscle cell health and function. Therefore, reducing the inflammatory response and cell death by targeting the STING pathway may help to treat skeletal muscle-related diseases. Drug development targeting STING would have a significant impact on the treatment of skeletal muscle-related disorders. In recent studies, several STING inhibitors have shown potential therapeutic effects in skeletal muscle-related disorders on osteoporosis, IVDD, OA, and other musculoskeletal diseases by improving bone and muscle homeostasis regulation, inhibiting ECM degradation, and reducing inflammation.

STING is a promising target for cancer, autoimmune diseases, and degenerative disorders. While agonists have been extensively developed and are entering clinical stages, the development of STING inhibitors is still in its early stage. The development of inhibitors faces many challenges. First, STING is a protein that is widely expressed in cells and plays different roles in different tissues and cell types, so the development of STING inhibitors needs to consider the issue of tissue specificity to avoid unwanted side effects and damage to healthy cells. Second, most STING inhibitors currently require effective delivery to target cells or tissues, such as by injection, and therefore better delivery systems need to be developed to improve the stability and bioavailability of the inhibitors *in vivo (* 217). Third, STING, as a key factor in the immune system, is closely related to immunomodulation, infection and cancer, and therefore targeted inhibitors of STING need to undergo complex biological evaluation and validation of their effects in different models to ensure the impact and safety on the whole immune system. Finally, the therapeutic efficacy and safety of STING inhibitors are the focus of development and require intensive clinical trials and evaluations to determine the optimal dose and treatment regimen to ensure therapeutic efficacy and safety.

In summary, STING is a complex player in various diseases, and the fine regulation of its signaling intensity is crucial in treating these diseases. The development of STING inhibitors shows great potential in treating autoimmune diseases and degenerative disorders associated with aging, and the use of novel techniques such as PROTACs, AUTOTACs, and artificial intelligence may lead to the development of more effective drugs in the future.

## Author contributions

CS and ZH wrote and reviewed the manuscript. HB, JL, XZ and QZ participated in organizing literatures and drawing figures. HY, TL, and YL reviewed and proofread the manuscript. All authors provided substantial contributions to this review, drafted and critically revised the manuscript, and designed and created the figures. All authors contributed to the article and approved the submitted version.
